# Multi-Functional Desaturases in Two *Spodoptera* Moths with ∆11 and ∆12 Desaturation Activities

**DOI:** 10.1007/s10886-019-01067-3

**Published:** 2019-04-02

**Authors:** Yi-Han Xia, Ya-Nan Zhang, Bao-Jian Ding, Hong-Lei Wang, Christer Löfstedt

**Affiliations:** 1 Department of Biology, Lund University, Sölvegatan 37, SE-223 62 Lund, Sweden; 2 College of Life Sciences, Huaibei Normal University, Dongshan Road 100, Huaibei, CN-235000 China

**Keywords:** *Spodoptera exigua*, *Spodoptera litura*, Pheromone biosynthesis, ∆11 desaturation, ∆12 desaturation

## Abstract

The beet armyworm, *Spodoptera exigua*, uses (*Z*,*E*)-9,12-tetradecadienyl acetate as the major component of its sex pheromone. Previous isotope-labeling experiments demonstrated an unusual ∆12 desaturase activity involved in the biosynthesis of this compound; however, the putative ∆12 desaturase gene has not been reported to date. In the present study, we confirmed this ∆12 desaturation pathway by in vivo labeling experiments, and characterized candidate desaturase genes in a yeast heterologous expression system. We demonstrated that a pheromone gland-specific desaturase, SexiDes5, uses palmitic acid and the subsequently chain-shortened product (*Z*)-9-tetradecenoic acid as substrates to produce (*Z*)-11-hexadecenoic and (*Z*,*E*)-9,12-tetradecadienoic acids, respectively. In addition, the homologous desaturase SlitDes5 from the congeneric *Spodoptera litura* had analogous functions.

**Electronic supplementary material:**

The online version of this article (10.1007/s10886-019-01067-3) contains supplementary material, which is available to authorized users.

## Introduction

Female moths emit species-specific sex pheromone blends that attract conspecific males over long distances. Approximately 75% of known moth sex pheromone components are C_10_-C_18_ monounsaturated or diunsaturated acetates, alcohols or aldehydes (Löfstedt et al. 2016). The biosynthetic pathways for this type of pheromone start from de novo synthesis of palmitic and stearic acids, followed by desaturation and limited chain shortening or elongation, to form the required carbon chain skeletons, before final modification of the terminal carboxyl group (Ando et al. 2004; Tillman et al. 1999; Wyatt 2003).

Fatty acid desaturases that introduce double bonds in specific positions of carbon chains are the most well studied enzymes involved in moth sex pheromone biosynthesis. These enzymes contain up to four transmembrane domains and three histidine-boxes (Shanklin et al. 1994). To date, a number of desaturases have been identified from moth pheromone glands, a specialized tissue where pheromones are produced. Different desaturases catalyze desaturation reactions that result in the introduction of double bonds in different positions and geometry (Arsequell et al. 1990; Bjostad and Roelofs 1983; Bjostad and Roelofs 1981; Foster and Roelofs 1996; Foster and Roelofs 1988; Löfstedt and Bengtsson 1988; Martinez et al. 1990; Zhao et al. 1990). Genes encoding corresponding desaturases have been characterized via heterologous expression systems, e.g., ∆5 desaturases in *Ctenopseustis obliquana* and *C. herana* (Hagström et al. 2014), a ∆6 desaturase in *Antheraea pernyi* (Wang et al. 2010), several ∆9 desaturases from a range of moth species (Liu et al. 2004; Liu et al. 2002; Rodríguez et al. 2004; Rosenfield et al. 2001), a ∆10 desaturase in *Planotortrix octo* (Hao et al. 2002), a ∆11 desaturase in *Trichoplusia ni* (Knipple et al. 1998), a ∆11/∆13 multifunctional desaturase in *Thaumetopoea pityocampa* (Serra et al. 2007), ∆14 desaturases in *Ostrinia* species (Roelofs et al. 2002), and a terminal desaturase in *Operophtera brumata* (Ding et al. 2011).

An unusual Δ12 desaturase activity involved in the formation of (*Z*,*E*)-9,12-tetradecadienoic acid (Z9,E12–14:acid) from (*Z*)-9-tetradecenoic acid (Z9–14:acid) was previously demonstrated in the almond moth, *Cadra cautella*, and the beet armyworm, *Spodoptera exigua* (Jurenka 1997, 2003), both using (*Z*,*E*)-9,12-tetradecenoic acetate (Z9,E12–14:OAc) as the major sex pheromone component (Brady et al. 1972; Kuwahara et al. 1971). In vivo labeling experiments in *Plodia interpunctella,* which also uses Z9,E12–14:OAc as a sex pheromone component, suggested the same pathway, i.e., ∆11 desaturation of palmitic acid yielding (*Z*)-11-hexadecenoic acid (Z11–16:acid), which is chain shortened to Z9–14:acid, with ∆12 desaturation producing Z9,E12–14:acid (Tsfadia et al. 2008). However, in another *Spodoptera* species, *S. littoralis*, which uses (*Z*,*E*)-9,11-tetradecenoic acetate (Z9,E11–14:OAc) as the major pheromone component, a Δ11 desaturase was postulated to act on the Z9–14:acid precursor to produce the pheromone compound (Dunkelblum and Kehat 1987).

To date, a gene encoding a Δ12 desaturase in a moth pheromone biosynthesis system has yet to be identified. In the present study, we performed in vivo labeling experiments with deuterium-labeled fatty acid precursors to confirm the Δ12 desaturation step in *S. exigua*, and functionally characterized, using a yeast expression system, the desaturase genes that perform these processes in *S. exigua* and *S. litura*.

## Materials and Methods

### Insects

Eggs of *S. exigua* were purchased from Keyun Biocontrol (Jiyuan, China). Upon hatching, larvae were reared at 23 ± 1 °C under a 17:7 L:D photoperiod and 70% RH, and were fed on a modified artificial diet based on the formula of Hinks and Byers (1976), using white beans instead of pea beans. Small amounts of propionic acid, sodium benzoate, vitamin mix and Wesson salt mixture were added for diet optimization. Male and female pupae were maintained separately. Adult females were fed with a 10% honey solution and used when 2–3-d-old.

### Chemicals

[14,14,14-^2^H_3_] Tetradecanoic acid (D_3_–14:acid) and [16,16,16-^2^H_3_] hexadecanoic acid (D_3_–16:acid) were purchased from Larodan AB (Malmö, Sweden). (*Z*)-11-[13,13,14,14,15,15,16,16,16-^2^H_9_] Hexadecenoic acid (D_9_-Z11–16:acid) was synthesized as described in Löfstedt et al. (1994), and (*Z*)-9-[11,11,12,12,13,13,14,14,14-^2^H_9_] tetradecenoic acid (D_9_-Z9–14:acid) was synthesized as described in Zhu et al. (1995). (*E*)-12-[14,14,14-^2^H_3_] tetradecenoic acid (D_3_-E12–14:acid) and Z9–14:acid were obtained from Prof. Cheng-Hua Zhao as gifts. Other fatty acids and synthetic pheromone components were available in our laboratory collection of compounds and were of various origins.

### Labeling Experiments and Sample Extraction

The deuterium-labeled precursors D_3_–16:acid, D_3_–14:acid, D_3_-E12–14:acid, D_9_-Z9–14:acid, D_9_-Z11–16:acid, as well as unlabeled Z9–14:acid, were dissolved in dimethylsulphoxide (DMSO) at 40 μg/μl. About 1 h into the scotophase, 0.4 μl of a solution was topically applied to the pheromone gland of a female. The same volume of DMSO was applied as a control to another group. After a 5 h incubation period, pheromone glands were excised, with five glands pooled in a 250 μl insert in a 1.5 ml glass vial to which 20 μl heptane was added. The glands were allowed to extract at room temperature for 30 min and the solvent, containing the pheromone components, was transferred to a new vial. The remaining lipids in the glandular residue were subsequently extracted with 100 μl chloroform:methanol (2:1 v:v) at room temperature overnight in order to analyze the fatty acyl precursors. This was accomplished by base methanolysis, which converted fatty acyl moieties to methyl esters (Bjostad and Roelofs 1984).

### Gas Chromatography/Mass Spectrometry (GC/MS)

Pheromone gland extracts and methyl esters of fatty acyl moieties were analyzed using an Agilent 5973 mass-selective detector coupled to an Agilent 6890 series gas chromatograph, equipped with either a polar (HP-INNOWax, 30 m × 0.25 mm, 0.25 μm film thickness) or non-polar column (HP-5MS, 30 m × 0.25 mm, 0.25 μm film thickness). Helium was the carrier gas.

For analyzing pheromone gland extracts, the oven temperature was set at 80 °C for 1 min, then increased to 190 °C at 10 °C.min^−1^, held for 10 min, and finally increased to 230 °C at 4 °C.min^−1^ and held for 10 min. Deuterium label incorporation into the pheromone components was detected using selected ion monitoring (SIM): *m/z* 61, 192 and 210 for Z9,E12–14:OAc and Z9,E11–14:OAc, *m/z* 61, 195 and 213 for D_3_-Z9,E12–14:OAc and D_3_-Z9,E11–14:OAc, *m/z* 61 and 194 for (*Z*)-9-tetradecenyl acetate (Z9–14:OAc), *m/z* 31 and 194 for (*Z*)-9-tetradecenol (Z9–14:OH), *m/z* 61 and 197 for D_3_-Z9–14:OAc, *m/z* 31 and 197 for D_3_-Z9–14:OH, *m/z* 61, 194 and 203 for D_9_-Z9–14:OAc, *m/z* 31, 194 and 203 for D_9_-Z9–14:OH, *m/z* 61 and 222 for (*Z*)-11-hexadecenyl acetate (Z11–16:OAc), *m/z* 61 and 225 for D_3_-Z11–16:OAc, *m/z* 31 and 222 for (*Z*)-11-hexadecenol (Z11–16:OH), and *m/z* 31 and 225 for D_3_-Z11–16:OH.

For fatty acid methyl esters (FAMEs), the oven temperature was set at 80 °C for 1 min, then increased to 230 °C at 10 °C.min^−1^ and held for 10 min. Deuterium label incorporation into putative pheromone precursors was detected by characteristic ions using SIM: *m/z* 242 and 245 for methyl myristate (14:Me) and D_3_–14:Me, respectively, *m/z* 208 and 240 for monounsaturated 14:Me, *m/z* 211 and 243 for monounsaturated D_3_–14:Me, *m/z* 164, 206 and 238 for methyl (*Z*,*E*)-9,12-tetradecadienoate (Z9,E12–14:Me), *m/z* 164, 209 and 241 for D_3_-Z9,E12–14:Me, *m/z* 236 and 268 for methyl (*Z*)-11-hexadecanoate (Z11–16:Me), *m/z* 239 and 271 for D_3_-Z11–16:Me, *m/z* 234 and 266 for diunsaturated methyl palmitate (16:Me), *m/z* 237 and 269 for diunsaturated D_3_–16:Me.

For analysis of yeast extracts, the oven temperature was set to 80 °C for 1 min, then increased at 10 °C.min^−1^ to 210 °C, held for 15 min, and then increased at 10 °C.min^−1^ to 230 °C for 20 min. Full scan mode was used to record mass spectra and compounds were identified by comparison of retention times and mass spectra with those of reference compounds.

DMDS derivatization (Dunkelblum et al. 1985; Vincent et al. 1987) was performed to determine the position of double bonds in target compounds, with the DMDS-adducts analyzed by GC/MS using a non-polar column (HP-5MS) with the following column oven program: 80 °C for 2 min, then increased at 15 °C.min^−1^ to 140 °C, then increased at 5 °C.min^−1^ to 260 °C, and held for 30 min.

### Sequences and Phylogenetic Analyses

Desaturase sequences were obtained from pheromone gland transcriptome databases for *S. exigua* (Zhang et al. 2017) and *S. litura* (Zhang et al. 2015). A phylogenetic tree was constructed for desaturases from *S. exigua* (Zhang et al. 2017) and *S. litura* (Zhang et al. 2015), based on a dataset containing 3 amino acid sequences from *S. exigua*, 1 amino acid sequence from *S. litura* and 53 amino acid sequences from other insects (Table S2). Evolutionary history was inferred by using the maximum likelihood method based on the JTT matrix-based model (Jones et al. 1992). The bootstrap consensus tree inferred from 500 replicates (Felsenstein 1985) is taken to represent the evolutionary history of the genes analyzed. Branches corresponding to partitions reproduced in less than 50% bootstrap replicates are collapsed. The initial tree for the heuristic search was obtained automatically by using BIONJ method with MCL distance matrix. All positions with less than 95% site coverage were eliminated, i.e., fewer than 5% alignment gaps, missing data, and ambiguous bases were allowed at any position. There was a total of 305 positions in the final dataset. Evolutionary analyses were conducted in MEGA5 (Tamura et al. 2011).

### Cloning of Desaturases cDNA

Twenty pheromone glands of 1–4-d-old female *S. exigua* and *S. litura* for each extraction were dissected between 5 and 7 h into the scotophase, respectively. For each species, the total RNA from abdominal tissue without pheromone gland was dissected from two females as a control. Total RNA was extracted using the Trizol® reagent (Invitrogen), and DNaseI digestion was included to avoid genomic DNA contamination. The quality of total RNA was checked with a spectrophotometer (NanoDropTM 2000, Thermo Fisher Scientific, USA). The single-stranded cDNA templates were synthesized from 1 μg of total RNA from *S. exigua* and *S. litura* pheromone gland and abdomen tissue samples using the PrimeScript RT Master Mix (Thermo Scientific™).

The desaturase-like SexiDes5 (GenBank accession number: KU755471), SlitDes5 (GenBank accession number: XP_022827905.1), SexiDes7 (GenBank accession number: AFO38465.1) and SexiDes11 (GenBank accession number: AFO38464.1), previously described to show pheromone gland-biased expression (Zhang et al. 2017), were amplified by PCR on a Veriti Thermo Cycler with specific gene primers (Table S1) under the following conditions: 95 °C for 5 min, 35 cycles of 95 °C for 30 s, 55 °C for 30 s, 72 °C for 40 s, followed by a final extension for 10 min at 72 °C. The reactions were performed in a total volume of 50 μl, containing 25 μl of Maxima polymerase master mix (Thermo Scientific™), 2.5 μl for each primer (10 μM) (Table S1), 1 μl of sample cDNA (15 ng/μl), and 19 μl of sterilized H_2_O. The PCR products were analyzed by electrophoresis on 1.5% *w*/*v* agarose gel in TAE buffer (40 mM Tris-acetate, 2 mM Na_2_EDTA-H_2_O) and were then gel purified with a GeneJET Gel Extraction Kit (Thermo Scientific™), cloned into pDONR221 by BP reaction to generate entry clone for each gene (Gateway cloning system, Invitrogen) and sequenced by M13+ and M13- primers. The correct entry clones were cloned into destination vector pYEX-CHT-DEST (Ding and Löfstedt 2015) by LR reaction to generate the expression clone for each gene. The expression clones were sequenced with pYEX-CHT vector primers (Table S1) using the BigDye Terminator cycle sequencing kit v1.1 followed by analysis on an in-house capillary ABI 3100 sequencer instrument (Applied Biosystems).

### Functional Assay of SexiDes5, SexiDes7, SexiDes11 and SlitDes5

The presence and directionality of SexiDes5, SexiDes7, SexiDes11 and SlitDes5 were verified by sequencing with the vector-specific primers pYEX-CHT-F and pYEX-CHT-R, then the sequence-verified expression clones of SexiDes5-pYEX-CHT, SexiDes7-pYEX-CHT, SexiDes11-pYEX-CHT and SlitDes5-pYEX-CHT were transformed into *∆ole1/∆elo1 Saccharomyces cerevisiae* yeast strain (Schneiter et al. 2000) using the S.c. easy yeast transformation kit (Thermo Scientific™), and were selected at 30 °C for uracil and leucine prototrophs on SC-ura-leu plates containing 2% (*w*/*v*) glucose, 0.7% YNB (yeast nitrogen base, without amino acids, with ammonium sulfate), synthetic complete amino acid drop-out lacking uracil and leucine (Formedium, Norwich, England), 1% tergitol (type NP-40, Sigma-Aldrich), 0.01% adenine (Sigma-Aldrich) and 0.5 mM oleic acid (Sigma-Aldrich) for a week. For the functional assay, individual yeast colonies were inoculated in 5 ml of the same selective liquid medium for 48 h at 30 °C and 300 rpm, diluted to an OD600 of 0.4 in 250 ml flasks containing 20 ml selective medium and 2 mM CuSO_4_ and added supplementation of a FAME precursor (i.e., 14:Me, Z9–14:Me or Z11–16:Me; Larodan Fine Chemicals, Malmö, Sweden). Each FAME precursor was prepared at a concentration of 100 mM in 96% ethanol and added to reach a final concentration of 0.5 mM in the culture medium (Liénard et al. 2010). Afterwards, yeasts were grown for 48 h at 30 °C and 300 rpm. Lipids were extracted from yeast pellets with 500 μl chloroform/methanol (2:1 v:v) at room temperature for 1 h, followed by base-methanolysis and transesterification as previously described (Dunkelblum and Kehat 1987) to convert all fatty acids to their corresponding methyl esters. The products were recovered in *n*-heptane prior to GC/MS analysis. The position of double bonds in monoenes was determined by DMDS derivatization (Dunkelblum et al. 1985) followed by GC/MS analysis.

## Results

### Confirmation of the Δ12-Pathway by In Vivo Labeling

**Fig. 1:**
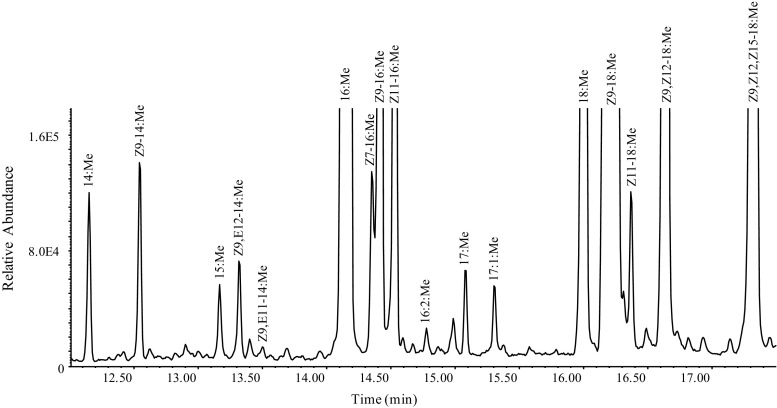
**Fatty acid profile of*Spodoptera exigua*female pheromone glands.** Gas chromatography/mass spectrometry analysis of fatty acids in *S. exigua* pheromone gland as methyl esters. 16:2:Me and 17:1:Me refer to a diunsaturated 16 carbon methyl ester and a monounsaturated 17 carbon methyl ester, respectively, each of unknown double bond position/geometry

A number of C_14_ and C_16_ acetates and alcohols, including saturated hexadecyl acetate (16:OAc) and hexadecanol (16:OH,) monounsaturated Z9–14:OAc, Z9–14:OH, Z11–16:OAc and Z11–16:OH, and doubly unsaturated Z9,E12–14:OAc and (*Z*,*E*)-9,12-tetradecadienol (Z9,E12–14:OH), were found in gland extracts of *S. exigua* based on GC/MS analysis. In addition to various ubiquitous fatty acids, including myristic, palmitic, palmitoleic, stearic, oleic, linoleic and linolenic acids, the following fatty acyl compounds (analyzed as FAMEs) were found in the pheromone gland extracts in relatively abundant amounts: monounsaturated Z11–16:acid and Z9–14:acid, and a doubly unsaturated Z9,E12–14:acid. In addition to these acids, traces of (*Z*,*E*)-9,11-tetradecadienoic acid (Z9,E11–14:acid) and an unidentified doubly unsaturated C_16_ acid were detected, but no monounsaturated tetradecenoic or hexadecenoic acids with a double-bond at Δ12 or Δ14 locations were detected in the present study (Fig. 1).

**Fig. 2:**
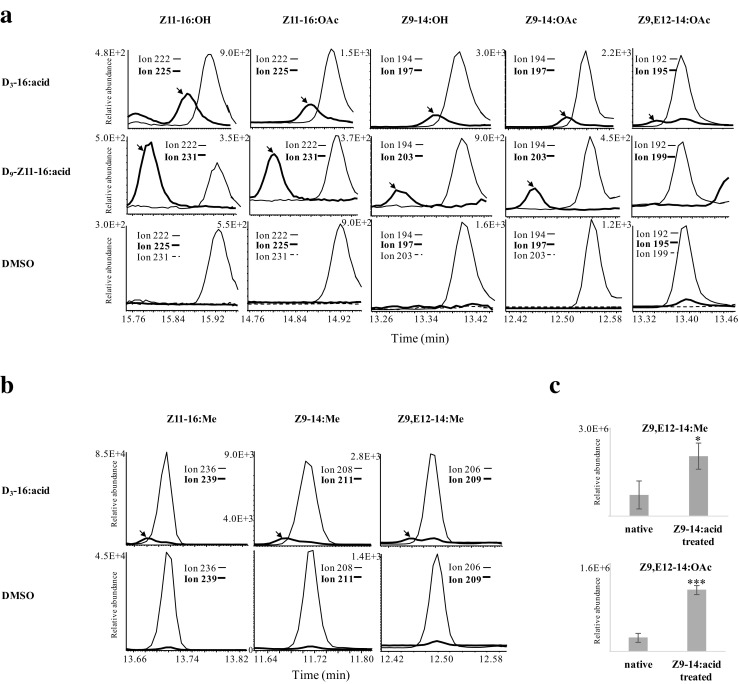
**Incorporation of deuterium labels into pheromone gland components and fatty acyl precursors in*Spodoptera exigua*. a.** Label incorporation from [16,16,16-^2^H_3_] hexadecanoic acid (D_3_–16:acid) and (*Z*)-11-[13,13,14,14,15,15,16,16,16-^2^H_9_] hexadecenoic acid (D_9_-Z11–16:acid) in comparison with that from a DMSO control. The *m/z* 222, 194 and 192 together with reference *m/z* 31 (for alcohols) and 61 (for acetates) were used to monitor native (*Z*)-11-hexadecenol (Z11–16:OH), (*Z*)-11-hexadecenyl acetate (Z11–16:OAc), (*Z*)-9-tetradecenol (Z9–14:OH), (*Z*)-9-tetradecenyl acetate (Z9–14:OAc) and (*Z*,*E*)-9,12-tetradecenoic acetate (Z9,E12–14:OAc), respectively. The *m/z* 225, 197 and 195 (indicated by arrows) demonstrate incorporation of three deuterium atoms when D_3_–16:acid was applied, while *m/z* 231, 203 and 199 demonstrate incorporation of nine or seven deuterium atoms when D_9_-Z11–16:acid was applied. **b.** Label incorporation from D_3_–16:acid into (*Z*)-11-hexadecenoic acid (Z11–16:acid), (*Z*)-9-tetradecenoic acid (Z9–14:acid) and (*Z*,*E*)-9,11-tetradecadienoic acid (Z9,E11–14:acid). The *m/z* 236, 208 and 206 were used to monitor native methyl (*Z*)-11-hexadecanoate (Z11–16:Me), methyl (*Z*)-9-tetradecanoate (Z9–14:Me) and methyl (*Z*,*E*)-9,12-tetradecanoate (Z9,E12–14:Me), respectively, while *m/z* 239, 211 and 209 were used to monitor the incorporation of three deuterium atoms (from applied acid) into these compounds. **c.** Amounts of Z9,E12–14:Me and Z9,E12–14:OAc in gland extracts before and after topical application of Z9–14:acid (SPSS v.20, independent samples t-test, *P < 0.05, ***P < 0.001, *n* = 6)

Deuterium-labeled D_3_–16:acid, D_9_-Z11–16:acid, D_3_–14:acid, D_9_-Z9–14:acid and D_3_-E12–14:acid were topically applied to the pheromone glands of *S. exigua* to trace the biosynthetic pathway. The results showed that deuterium atoms from D_3_–16:acid were incorporated into all the detected acetates and alcohols, including Z11–16:OAc, Z11–16:OH, Z9–14:OAc, Z9–14:OH, Z9,E12–14:OAc and Z9,E12–14:OH (Fig. 2a), as well as into the corresponding FAMEs, i.e., Z11–16:Me, Z9–14:Me, Z9,E12–14:Me and 14:Me (Fig. 2b). Label from D_9_-Z11–16:acid was incorporated into Z11–16:OAc, Z11–16:OH, Z9–14:OAc, Z9–14:OH and the fatty acyl intermediate Z9–14:Me, but not into doubly unsaturated Z9,E12–14:OAc, Z9,E12–14:OH and the corresponding Z9,E12–14:Me (Fig. 2a). Similarly, label from D_9_-Z9–14:acid was incorporated into Z9–14:OAc, Z9–14:OH, but not into any doubly unsaturated compounds. In contrast, when D_3_–14:acid and D_3_-E12–14:acid were applied, label incorporation was not detected in any of the above components (Fig. S1). Notably, when non-labeled Z9–14:acid was applied to the pheromone gland, the amount of Z9,E12–14:OAc increased significantly (Fig. 2c).

### *Characterization of desaturase genes in *S. exigua and S. Litura

Reverse transcription-PCR was used to amplify desaturase candidate genes by their gene specific primers from the pheromone glands of *S. exigua* and *S. litura* cDNA library, respectively. SexiDes5 encoded protein with amino acid (aa) identity of 90% to ∆11 desaturase of *S. litura*, of which GenBank accession number was AGH12217.1. SexiDes7 and SexiDes11 encoded proteins with aa identity of 90% and 98% to Acyl-CoA desaturase 4 from *Helicoverpa armigera* (GenBank accession number: AKU76403.1) and ∆9 desaturase from *Spodoptera littoralis* (GenBank accession number: AAQ74258.1), respectively. For the desaturase candidate gene from *S. litura*, SlitDes5 shared 96% aa identity to the Acyl-CoA ∆11 desaturase of *S. littoralis* of which GenBank accession number was AY362879.

The four full-length sequences of SexiDes5 (1017 bp), SexiDes7 (1116 bp), SexiDes11 (1062 bp) and SlitDes5 (1017 bp) were obtained from the pheromone gland cDNA library transcripts (Zhang et al. 2017) and encoded proteins with 339 aa, 372 aa, 354 aa and 339 aa, respectively.

**Fig. 3:**
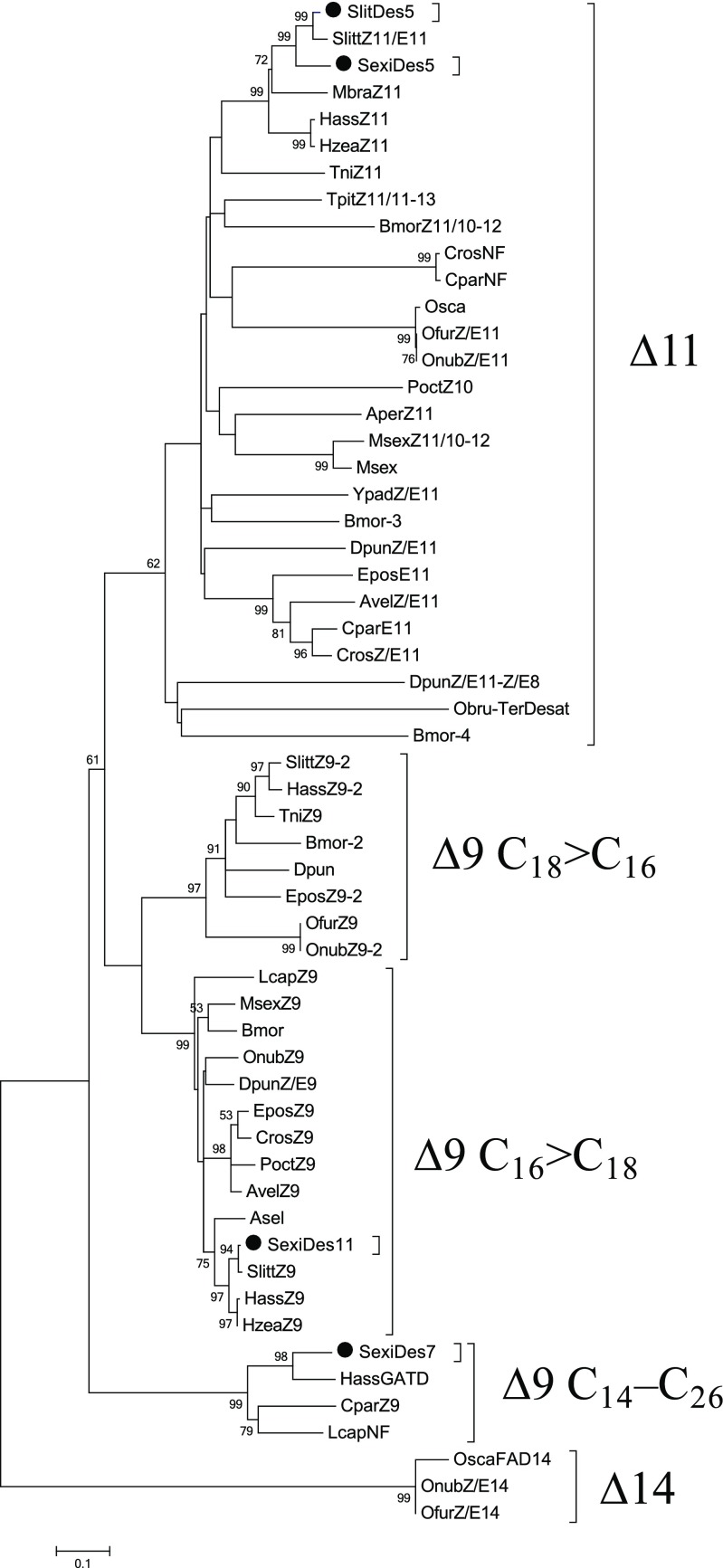
**Phylogeny of fatty acyl desaturases in moths and butterflies.** Evolutionary analyses were conducted in MEGA5 and the evolutionary history was inferred by using the maximum likelihood method based on the JTT matrix-based model. The bootstrap consensus tree inferred from 500 replicates is taken to represent the evolutionary history of the gene analyzed. Branches corresponding to partitions reproduced in less than 50% bootstrap replicates are collapsed. The predicted *Spodoptera exigua* (Sexi) and *S. litura* (Slit) fatty acyl desaturase orthologs are marked in round spots. Amino acid sequences of the species and their accession numbers are given in Table S2

The phylogenetic tree using desaturase protein sequences from *Clubiona parallela*, *Lampronia capitella*, *Ostrinia nubilalis*, *O. scapulalis*, *S. littoralis* and some other lepidopteran species (Fig. 3) showed that SexiDes5 and SlitDes5 clearly clustered into the clade of ∆11 desaturases, while SexiDes7 and SexiDes11 clustered in the clade of ∆9 (14C-26C) and ∆9 (16C > 18C) desaturases, respectively.

### Heterologous Expression of SexiDes5, SexiDes7, SexiDes11 and SlitDes5

**Fig. 4:**
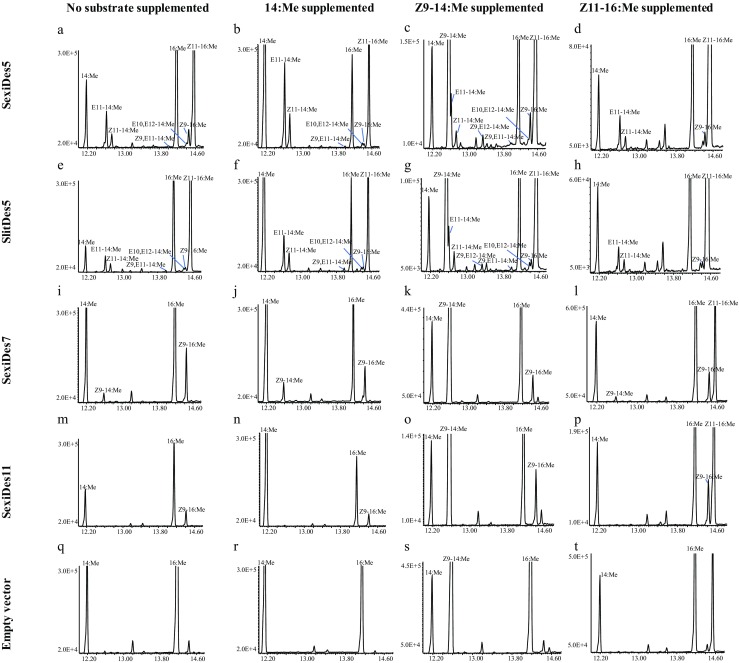
**Heterologous expression of candidate desaturases genes from*S. exigua*and*S. litura*.** Fatty acid methyl ester profiles of yeast expressing the SexiDes5 (**a**–**d**), SlitDes5 (**e**–**h**), SexiDes7 (**i**–**l**), SexiDes11 (**m**–**p**) and empty vector of pYEX-CHT (**q**–**t**). (**a**) SexiDes5 without substrate supplementary. (**b**–**d**) SexiDes5 supplemented with methyl myristate (14:Me), methyl (*Z*)-9-tetradecanoate (Z9–14:Me) or methyl (*Z*)-11-hexadecanoate (Z11–16:Me), respectively. (**e**) SlitDes5 without supplementary substrate. (**f**–**h**) SlitDes5 supplemented with 14:Me, Z9–14:Me or Z11–16:Me, respectively. (**i**) SexiDes7 without supplementary substrate. (**j**–**l**) SexiDes7 supplemented with 14:Me, Z9–14:Me or Z11–16:Me, respectively. (**m**) SexiDes11 without supplementary substrate. (**n**–**p**) SexiDes11 supplemented with 14:Me, Z9–14:Me or Z11–16:Me, respectively. (**q**) Yeast expressing empty vector without supplementary substrate. (**r**–**t**) Yeast expressing empty vector supplemented with 14:Me, Z9–14:Me or Z11–16:Me, respectively. Methyl (*Z*)-9-octadecanoate (Z9–18:Me) was supplemented as nutrition to all the incubations

The ORF of each desaturase (SexiDes5, SexiDes7, SexiDes11 and SlitDes5) was subcloned into the Cu^2+^ inducible vector pYEX-CHT, and functional expression was conducted in *ole1/elo1* deficient *Saccharomyces cerevisiae* yeast. After two days of incubation under copper induction and in the presence of supplemented 14:Me, Z9–14:Me or Z11–16:Me substrates, yeast cells were harvested and subjected to fatty acid analysis. GC/MS analyses showed that the yeast harboring SexiDes5 produced a relatively high amount of Z11–16:Me (approximately 20% of the total fatty acids), as well as the Δ11-monounsaturated E11–14: Me, Z11–14:Me and Z11–18:Me, both with and without above substrates added (Fig. 4a–d). SexiDes5 also produced trace amount of doubly unsaturated Z9,E11–14:Me and E10,E12–14:Me in all the samples except for the one with Z11–16:Me supplemented. Notably, when the yeast was supplemented with Z9–14:Me, a small amount of Z9,E12–14:Me was produced (Fig. 4c). However, this compound was neither detected from the samples supplemented with other substrates, i.e., 14:Me, Z11–16:Me, nor from the one without supplementary substrate (Fig. 4d). In the control yeast carrying an empty pYEX-CHT vector (Fig. 4q–t), or the ones expressing SexiDes11 and SexiDes7, the above-mentioned Δ11-monounsaturated and doubly unsaturated compounds were not produced, but for the later two cases, SexiDes11 produced Z9–16:Me (Fig. 4m–p) and SexiDes7 produced both Z9–14:Me and Z9–16:Me (Fig. 4i–l). Similar results to those obtained with SexiDes5 were found from the yeast expressing the homologous desaturase SlitDes5 (Fig. 4e–h).

## Discussion

**Fig. 5:**
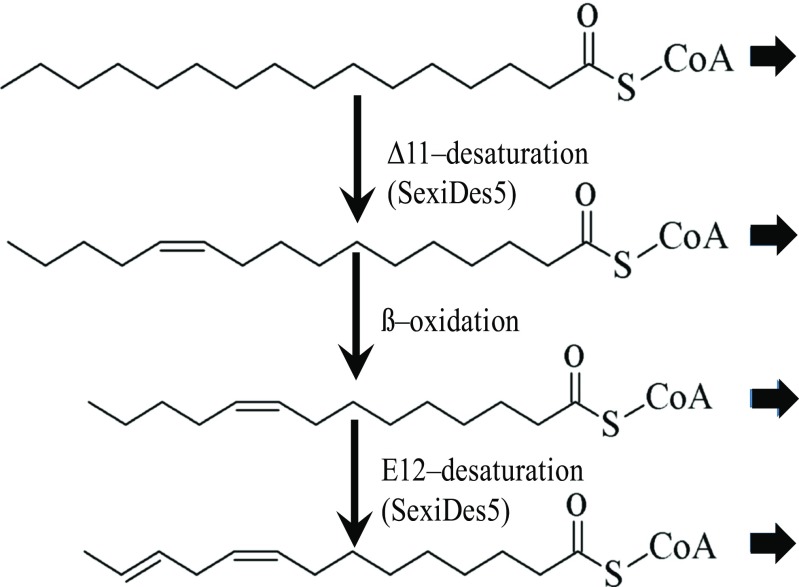
**Biosynthetic pathway for sex pheromone of*Spodoptera exigua*.** Proposed pathways for biosynthesis of the main pheromone component in *S. exigua*. The broad arrows indicate reduction and acetylation to form the alcohol and acetate ester pheromone components

The results from our in vivo labelling experiment support the previously proposed ∆12-pathway for the production of Z9,E12–14:OAc in *S. exigua* (Jurenka 1997). A ∆11 desaturase acts on palmitic acid to produce Z11–16:acid, which is then chain-shortened to Z9–14:acid, followed by the second desaturation at the ∆12 position to form Z9,E12–14:acid. This doubly unsaturated fatty acyl intermediate is reduced to the corresponding alcohol, and finally acetylated to the corresponding acetate (Fig. 5). The deuterium label from D_3_–16:acid was incorporated into both monounsaturated and doubly unsaturated pheromone components and the corresponding precursors; however, label from D_9_-Z11–16:acid or D_9_-Z9–14:acid was not incorporated into Z9,E12–14:OAc and Z9,E12–14:OH, possibly because of an isotope effect from the nine-deuterium atoms in the labeled precursors interfering with the ∆12 desaturase activity (Jurenka 1997). However, application of non-labeled Z9–14:acid increased the titer of Z9,E12–14:acid intermediate and the corresponding acetate, supporting Z9–14:acid being the immediate precursor of Z9,E12–14:acid.

In order to find part of the molecular mechanism for the pheromone production pathways in *S. exigua*, we characterized three desaturase candidate genes that had very high abundance in the transcriptome data of pheromone glands from *S. exigua* (Zhang et al. 2017). Among the candidate desaturases, SexiDes5 was the most abundant transcript in the pheromone gland (Zhang et al. 2017). This gene clusters in the ∆11 desaturase clade (Fig. 3) and has a pheromone gland-specific expression according to our RT-PCR results (Fig. S2). The abundance of SexiDes7 and SexiDes11 was not as high as for SexiDes5 and although both were reported to show pheromone gland-biased expression in a previous study (Zhang et al. 2017) we could not reproduce this result. Furthermore, SexiDes7 and SexiDes11 clustered in the clade of ∆9 (14C-26C) and ∆9 (16C > 18C) desaturases, respectively. We found no evidence for ∆9 desaturation being involved in pheromone biosynthesis in the two *Spodoptera* species.

In the heterologous expression system, SexiDes5 and its homolog SlitDes5 from *S. litura* inserted a ¨*cis*¨ double bond at the ∆11 position in palmitic acid, as well as in myristic and stearic acids. These deaturases can also produce doubly unsaturated compounds, including conjugated Z9,E11–14:acid and (*E*,*E*)-10,12-tetradecadienoic acid (E10,E12–14:acid), and non-conjugated Z9,E12–14:acid. It is interesting that SexiDes5 and SlitDes5 make a “*cis*” double bond in saturated 16C and 18C substrates but both “*cis*” and “*trans*” double bonds in 14C substrates. The difference in chain length and structural difference between saturated and monounsaturated substrates might influence the acyl chain entry into the ¨kink¨ in the substrate-binding tunnel. These differences between substrates and the geometry of the tunnel might influence the regioselectivity and stereospecificity of the desaturation reaction (Bai et al. 2015). It is worth mentioning that the homologous desaturase SlittZ/E11 from *S. littoralis* that shares 89.3% and 96.4% aa sequence similarity with SexiDes5 and SlitDes5, respectively, was also found to produce both monounsaturated Z11–16:acid and doubly unsaturated 10,12-tetradecadienoic and 10,12-hexadecadienoic acids in yeast without substrate supplementation (Serra et al. 2006). For *S. exigua*, the production of the Z9,E12–14:acid precursor is a key step in the pheromone biosynthetic pathway, whereas, for *S. litura* and *S. littoralis* the production of Z9,E11–14:acid should be crucial since both use Z9,E11–14:OAc as the major sex pheromone component. Similar to SexiDes5, SlitDes5 has a pheromone gland-specific expression and is the most abundant desaturase transcript in the pheromone gland transcriptome (Zhang et al. 2015), suggesting that it is likely a key desaturase involved in pheromone production in *S. litura*. The two homologs SexiDes5 and SlitDes5 performed the same function under in vitro condition, but in the pheromone gland of the respective species (i.e., in vivo conditions) there might be additional mechanisms to ensure the directed biosynthesis of the Z9,E12- and Z9,E11–14:acid precursors and corresponding pheromone components.

In two other insect species, *Acheta domesticus* and *Tribolium castaneum,* ∆12 desaturases with ancestral ∆9 desaturase origin have been found. These desaturases are, however, not involved in sex pheromone biosynthesis but in the production of linoleic acid (Zhou et al. 2008). As for the production of Z9,E12–14:OAc, Antony et al. (2015) proposed an alternative pathway in *Ephestia cautella* involving a combination of Δ11 and Δ14 desaturation of palmitic acid followed by chain-shortening. However, this pathway is unlikely in *S. exigua* because 1) the mass spectrum of the DMDS adduct of the minor doubly unsaturated C_16_ acid in the pheromone gland of *S. exigua* exhibited *m/z* 84, 87, 189, 277 and 360, implying the positions of the two double bonds at ∆7 and ∆10, possibly through chain-shortening product of linoleic acid, 2) no monounsaturated hexadecenoic acids with a double-bond at C14 were found in the pheromone gland, 3) no deuterium label was incorporated from D_3_–16:acid into the above-mentioned hexadecadienoic acid, and 4) no Z11,E14–16 or Z9,E12–14 compound was observed after supplementing the SexiDes5-pYEX-CHT yeast with Z11–16:Me.

In conclusion, we report that the multi-functional desaturase homologs SexiDes5 and SlitDes5 are active in sex pheromone biosynthesis in two *Spodoptera* species, *S. exigua* and *S. litura*. They have a high ∆11 desaturase activity on palmitic acid, and they are also able to catalyze production of the doubly-unsaturated pheromone precursors Z9,E12–14:acid and Z9,E11–14:acid by interaction with Z9–14:acid in the respective species.

## Electronic supplementary material

ESM 1 (DOCX 144 kb)
