# Supplementary material for: Multi-Functional Desaturases in Two Spodoptera Moths with ∆11 and ∆12 Desaturation Activities
Source: J Chem Ecol. 2019 Apr 2;45(4):378–87. doi: 10.1007/s10886-019-01067-3 (PMC6476860; doi:10.1007/s10886-019-01067-3)
Supplement: Supplementary file 1 — (DOCX 144 kb) [file 10886_2019_1067_MOESM1_ESM.docx]

Multi-functional desaturases in two *Spodoptera* moths that have ∆11 and ∆12 desaturation activities

Yi-Han Xia^1^*, Ya-Nan Zhang^2^, Bao-Jian Ding^1^, Hong-Lei Wang^1^, Christer Löfstedt^1^

1 Department of Biology, Lund University, Sölvegatan 37, SE-223 62 Lund, Sweden.

2 College of Life Sciences, Huaibei Normal University, Dongshan Road 100, CN-235000, Huaibei, China

* Corresponding author. Phone: +46 46 222 4498

Email address: [yihan.xia@biol.lu.se](mailto:yihan.xia@biol.lu.se)

**Figure legends**

**Figure S1.** Partial GC/MS chromatograms (selected ion monitoring) of pheromone gland extracts obtained from female *S. exigua*after topical application of [14,14,14-^2^H_3_] tetradecanoic acid (D_3_-14:acid) (a) and (*E*)-12-[14,14,14-^2^H_3_] tetradecenoic acid (D_3_-E12-14:acid) (b). D_3_-labelled specimens should elute approximately 6 s before the native compound. Thus these four panels do not indicate any incorporation of the labelled precursors into the respective pheromone compounds.

**Figure S2**. Expression of SexiDes5 and glyceraldehyde-3-phosphate dehydrogenase (GAPDH) in pheromone glands (PG) and abdomen (Ab) of *S. exigua*, determined by semi-quantitative RT-PCR. Amplification without template was used as negative control. Amplicon sizes: SexiDes5, 237bp; SexiGAPDH, 150bp.

**Figure S1.**

**Figure S2.**

**Table S1.** Primers used in this study.

| Gene abbreviation | Primer sequence (5’-3’) |
| --- | --- |
| SexiDes5_F  SexiDes5_R  SexiDes7_F  SexiDes7_R  SexiDes11_F  SexiDes11_R  SlitDes5_F  SlitDes5_R  pYEX-CHT_F  pYEX-CHT_R  SexiDes5_210F  SexiDes5_448R  GAPDH_F  GAPDH_R | GGGGACAAGTTTGTACAAAAAAGCAGGCTTAATGGCTCAAACTATACAGAC  GGGGACCACTTTGTACAAGAAAGCTGGGTTTTATTCACCTTTTATATTTTTCAAGTTC  GGGGACAAGTTTGTACAAAAAAGCAGGCTTAATGGCCGCAATGTCAGGAGCCCCGCTA  GGGGACCACTTTGTACAAGAAAGCTGGGTTCTATTCAGTTTTTCTGTGGAC  GGGGACAAGTTTGTACAAAAAAGCAGGCTTAATGGCTCCAAATATATCGGAAGAC  GGGGACCACTTTGTACAAGAAAGCTGGGTTTTAATCATCCTTAGGATTAATCCTAATAGC  GGGGACAAGTTTGTACAAAAAAGCAGGCTTAATGGCGCAATGTGTACAAACAACAACGAT  GGGGACCACTTTGTACAAGAAAGCTGGGTTCTATTCGCCTTTTGTATTTTTTAAATCCTCTGGCGTATC  CATATAGAAGTCATCGA  TTTGCAGCTACCACATT  GTTTGTTATAGCAGAAATAGGAATC  CAAATAACCAGCCGATGTGTG  GACAACCACTCATCTATCTTCG  AACATTTATCTCTACAACGCAATC |

**Table S2** Accession numbers for amino acid sequences of desaturases phylogenetic analyses.

| **Species** | **Gene name** | **Acc. number** |  |
| --- | --- | --- | --- |
| *S. exigua* | SexiDes5 | KU755471 |  |
| *S. exigua* | SexiDes7 | AFO38465.1 |  |
| *S. exigua* | SexiDes11 | AFO38464.1 |  |
| *S. litura* | SlitDes5 | XP_022827905.1 |  |
| *O. nubilalis* | OnubZ/E14 | AAL35330 |  |
| *O. nubilalis* | OfurZ/E14 | AAL35330 |  |
| *O. scapulalis* | OscaFAD14 | BAE97679 |  |
| *C. parallela* | CparZ9 | AAQ12887 |  |
| *H. assulta* | HassGATD | AAM28480 |  |
| *L. capitella* | LcapNF | ABX71629 |  |
| *L. capitella* | LcapZ9 | ABX71627 |  |
| *S. littoralis* | SlittZ9 | AAQ74258 |  |
| *H. assulta* | HassZ9 | AAM28481 |  |
| *A. selenaria cretacea* | Asel | BAF97042 |  |
| *P. octo* | PoctZ9 | AAF73073 |  |
| *A. velutinana* | AvelZ9 | AAF44709 |  |
| *E. postvittana* | EposZ9 | AAL35750 |  |
| *C. rosaceana* | CrosZ9 | AAN39697 |  |
| *O. nubilalis* | OnubZ9 | AAF44710 |  |
| *D. punctatus* | DpunZ/E9 | ABX71810 | |
| *M. sexta* | MsexZ9 | CAJ27975 | |
| *B. mori* | Bmor | NP_001036971 | |
| *H. zea* | HzeaZ9 | AAF81788 | |
| *T. ni* | TniZ9 | AAB92583 | |
| *S. littoralis* | SlittZ9-2 | AAQ74257 | |
| *H. assulta* | HassZ9-2 | AAM28484 | |
| *D. punctatus* | Dpun | ABX71813 | |
| *O. furnacalis* | OfurZ9 | AAL27034 | |
| *O. furnacalis* | OnubZ9-2 | AAL27034 | |
| *B. mori* | Bmor-2 | NP_001037018 | |
| *E. postvittana* | EposZ9-2 | AAK94070 | |
| *E. postvittana* | EposE11 | AAL11496 | |
| *A. velutinana* | AvelZ/E11 | AAL16642 | |
| *C. parallela* | CparE11 | AAQ12891 | |
| *D. punctatus* | DpunZ/E11 | ABX71809 | |
| *T. ni* | TniZ11 | AF035375 | |
| *M. brassicae* | MbraZ11 | ABX90049 | |
| *H. assulta* | HassZ11 | AF482908 | |
| *S. littoralis* | SlittZ11/E11 | AY362879 | |
| *T. pityocampa* | TpitZ11/11-13 | ABO43722 | |
| *O. scapulalis* | Osca | BAE66602 | |
| *O. furnacalis* | OfurZ/E11 | AAL32060 | |
| *O. nubilalis* | OnubZ/E11 | AAL35331 | |
| *C. rosaceana* | CrosNF | AAN39698 | |
| *Y. padellus* | YpadZ/E11 | ADP21588 | |
| *B. mori* | Bmor-3 | NP_001036914 | |
| *M. sexta* | MsexZ11/10-12 | CAJ27976 | |
| *B. mori* | BmorZ11/10-12 | AAG16901 | |
| *A. pernyi* | AperZ11 | ADO85596 | |
| *P. octo* | PoctZ10 | AAG54077 | |
| *O. brumata* | Obru-TerDesat | AEH95845 | |
| *D. punctatus* | DpunZ/E11-Z/E8 | ABX71808 | |
| *B. mori* | Bmor-4 | NP_001040141 | |
| *M. sexta* | Msex | CAJ43430 | |
| *H. zea* | HzeaZ11 | AAF81787 | |
| *C. rosaceana* | CrosZ/E11 | AAN41250 | |
| *C. parallela* | CparNF | AAN39693 | |
